# Bedeutung von CI-Nutzungsverhalten und CI-Anpassung für sprachproduktive Leistungen sehr früh cochleaimplantierter Kinder

**DOI:** 10.1007/s00106-020-00942-w

**Published:** 2020-09-15

**Authors:** C. Glaubitz, T. Liebscher, U. Hoppe

**Affiliations:** Hals-Nasen-Ohrenklinik, Kopf- und Halschirurgie, Cochlear-Implant-Centrum CICERO, Universitätsklinikum Erlangen, Waldstr. 1, 91054 Erlangen, Deutschland

**Keywords:** Cochleaimplantat, Datalogging, CI-Nutzung, Sprachentwicklung, Sprachproduktion, Cochlear implant, Data logging, CI use, Language development, Speech production

## Abstract

**Hintergrund:**

Das Alter bei Versorgung mit einem Cochleaimplantat (CI) nimmt bei Kindern wesentlich Einfluss auf deren Lautsprachentwicklung. Als weitere potenzielle Einflussfaktoren sollten das CI-Nutzungsverhalten und die Häufigkeit der CI-Anpassungen evaluiert sowie die frühkindlichen sprachproduktiven Leistungen sehr früh CI-versorgter Kinder dargestellt werden.

**Methodik:**

Die retrospektive Studie untersuchte 34 bilateral cochleaimplantierte Kinder mit einem CI-Versorgungsalter von *M* = 8,8 (*SD* = 1,7) Monaten. Im dritten Lebensjahr wurden die sprachproduktiven Leistungen, die Häufigkeit der CI-Anpassungen und das CI-Nutzungsverhalten anhand des systemintegrierten Dataloggings ausgewertet und in Bezug gesetzt.

**Ergebnisse:**

Etwa die Hälfte der Kinder erzielte lebensaltersgemäße sprachproduktive Leistungen. Die CI-Nutzungsdauer lag bei rund 8 h täglich. Zeitlich am längsten befanden sich die Kinder in ruhiger Umgebung, am kürzesten in Situationen mit Sprache im Störschall. Die tägliche CI-Nutzungsdauer erwies sich als signifikanter Prädiktor für die sprachproduktiven Leistungen; die Sprachexposition erwies sich als wesentlicher Prädiktor speziell für die Wortproduktion. Die Anzahl der täglichen Kontaktabbrüche der CI-Sendespule zum Implantat sowie die Anzahl der CI-Anpassungen pro Monat waren nicht mit den sprachproduktiven Leistungen korreliert.

**Schlussfolgerung:**

Sehr früh bilateral cochleaimplantierte Kinder können bereits im dritten Lebensjahr einen altersgemäßen Stand in der Sprachproduktion erreichen. Im Vergleich mit anderen Studien ist die tägliche CI-Nutzungsdauer als altersadäquat zu beurteilen. CI-Nutzungsdauer sowie die Dauer der täglichen Sprachexposition scheinen bedeutsamen Einfluss auf frühe Sprachkompetenzen zu nehmen. Diese Erkenntnisse sollten insbesondere in die prä- und postoperative Elternberatung einfließen.

## Abstract (en)

**Background:**

Children’s age at implantation is an important factor for their outcome in auditory and language skills with a cochlear implant (CI). CI use and frequency of CI fitting may also influence speech performance. Purpose of this study was to evaluate CI use and CI fitting of very early implanted infants and its potential influence on age-related speech production performance.

**Methods:**

Data of 34 bilaterally cochlea-implanted infants (age at CI in months: M = 8,8; SD = 1,7) were included. During the third year of life speech production performance was evaluated and related to datalogging-based CI use and number of CI fitting sessions.

**Results:**

About half of the cohort achieved speech production level within the normal range of hearing peers. Daily time of CI use was approximately 8 h. Analysis of listening environment showed that infants were exposed most of the time to quiet environment and least amount of time to speech in noise. Daily time of CI use seems to be a significant predictor of speech production, speech-exposition particularly predicts word production. Number of daily disconnection between CI-processor and implant as well as the monthly number of CI fitting sessions were not correlated with speech production.

**Conclusion:**

Very early cochlear implanted infants may achieve age-appropriate speech production performance in the third year of life. Time of daily CI use in the study cohort is comparable to results of other studies. Time of daily CI use and exposure to speech seem to be important factors for early speech production. These findings should be integrated in pre- and postoperative parent counselling.

## Hintergrund

Der Lautspracherwerb ist Teil eines komplexen Entwicklungsprozesses und stellt hörbeeinträchtigte Kinder vor besondere Herausforderungen. Eine Versorgung mit einem Cochleaimplantat (CI) bietet dabei an Taubheit grenzend schwerhörigen Kindern die Möglichkeit, sprachrelevanten akustischen Input zu erhalten, um auf dieser Basis Lautsprache als Kommunikationsmittel zu erwerben. Zahlreiche Faktoren können auf den Lautspracherwerb CI-versorgter Kinder begünstigend oder auch hemmend einwirken.

### CI-Versorgungsalter und CI-Nutzungsverhalten

Das Lebensalter des Kindes zum Zeitpunkt der CI-Versorgung und dessen Einfluss auf die späteren Sprachkompetenzen steht seit vielen Jahren im Zentrum pädagogisch-audiologischer Forschung. Durch eine frühzeitige CI-Versorgung wird die auditive Deprivationsphase minimiert und somit die Entwicklung der Lautsprache begünstigt. Vielfach belegt sind bessere Hör- und Sprachleistungen für ein CI-Versorgungsalter bis 24 Lebensmonate [9, 25]. Für 12 Monate und jünger ist die Befundlage derzeit noch inkonsistent [6], es scheint sich jedoch als begünstigender Einflussfaktor für die lautsprachliche Entwicklung zu bestätigen [10, 26]. Weitere Einflussfaktoren – wie psychosoziale Aspekte, präoperative Versorgung und zusätzliche Beeinträchtigungen – wurden ebenfalls diskutiert [4, 13, 26]. Auch das CI-Nutzungsverhalten sowie Einflüsse durch die Hörumgebung wurden betrachtet, jedoch stützten sich hier die Daten auf Angaben der Eltern [1]. Bei Hörgeräten ist es schon längere Zeit möglich, gespeicherte Nutzungsdaten (Datalogging) auszuwerten. Walker et al. [31] fanden, dass Eltern hörgeräteversorgter Kinder zu einer Überschätzung der täglichen Nutzungsdauer tendieren. Datalogging bietet daher eine Möglichkeit, Nutzungsverhalten objektiv abzubilden. Mit Einführung des Cochlear Nucleus 6 CI-Systems (Cochlear Ltd., Sydney, NSW, Australia) 2013 ist Datalogging nun auch für CI-Systeme verfügbar. Diese objektiv ermittelten Daten werden zunehmend im Rahmen von Studien ausgewertet.

### Aktuelle Befunde zum Datalogging

Eine multizentrische Studie von Oberhoffner et al. [27] ermittelte altersbezogene Referenzwerte für CI-Nutzungsdaten von CI-Trägern aller Altersgruppen. Bush et al. [8] untersuchten ebenfalls das CI-Nutzungsverhalten und fanden – neben einer hohen interindividuellen Variabilität – dass sich CI-Träger größtenteils in Umgebungen mit Störschall befinden. Den Zusammenhang zwischen täglicher CI-Nutzungsdauer und Sprachverstehen bei erwachsenen CI-Nutzern untersuchten Holder et al. [20] und fanden hohe Korrelationen. Hey et al. [19] zeigten anhand von Nutzungsanalysen erwachsener CI-Träger, dass die Sprachpegel am häufigsten zwischen 50 und 59 dB liegen und somit größte Alltagsrelevanz haben.

Studien zum dataloggingbasierten CI-Nutzungsverhalten bei Kindern finden sich aktuell nur wenige. Die genannten Studien von Bush et al. und Oberhoffner et al. ergaben für die Kleinkinder eine mediane tägliche CI-Nutzungsdauer von etwas mehr als 8 h. Wiseman und Warner-Czyz [33] fanden eine reduzierte tägliche CI-Nutzungsdauer (<8 h) bei etwa der Hälfte der untersuchten Kinder und identifizierten demografische und audiologische Variablen als Risikofaktoren für diese reduzierte CI-Nutzung. Auch Easwar et al. evaluierten die CI-Nutzung bei Kindern und deren Abhängigkeit von spezifischen Variablen sowie die Expositionszeiten in verschiedenen Hörumgebungen und Intensitätslevel [11]. In einer weiteren Studie [12] fanden Easwar et al. einen positiven Einfluss der CI-Nutzungsdauer auf perzeptive Sprachleistungen. Für sequenziell CI-versorgte Kinder zeigten die Autoren zudem, dass sich Asymmetrien im Sprachverstehen zwischen rechtem und linkem Ohr mit höherer täglicher CI-Nutzungsdauer reduzieren. Guerzoni und Cuda [17] ermittelten bei Kindern positive Zusammenhänge zwischen kumulierten CI-Nutzungszeiten bei Sprachexposition in Ruhe und im Störschall und auditiven sowie lexikalischen Kompetenzen.

### Erhebung frühkindlicher Sprachleistungen

Zur Erfassung frühkindlicher Sprachleistungen zählen zu den etablierten Verfahren im deutschsprachigen Raum der *Elternfragebogen zur Früherkennung von Risikokindern ELFRA‑2* [15] und der *Sprachentwicklungstest für zweijährige Kinder SETK‑2* [16]. Beide Verfahren eigenen sich zur Anwendung im Rahmen der postoperativen Rehabilitationsphase [22, 23] und ermöglichen eine Einschätzung der Sprachleistungen von CI-versorgten Kindern im Vergleich zu hörgesunden Normierungsstichproben. Für eine umfassende Beurteilung der frühkindlichen Sprachkompetenz sollten rezeptive und produktive Sprachbereiche bevorzugt separat betrachtet werden, insbesondere dann, wenn davon ausgehend adäquate Therapieziele formuliert werden. Die rezeptiven Fähigkeiten bilden in der Sprachentwicklung die Voraussetzung für die Produktion von Sprache [32]. Vor diesem Hintergrund erscheint eine Begrenzung auf Daten zur Sprachproduktion gerechtfertigt. Bisher gibt es keine Studien, die die Sprachproduktion und das CI-Nutzungsverhalten von Kindern in Bezug setzen.

### Fragestellungen der Studie

Die vorliegende Studie umfasst folgende Fragestellungen: Welche sprachproduktiven Leistungen zeigen Kinder im dritten Lebensjahr, die innerhalb des ersten Lebensjahrs bilateral mit CI versorgt wurden? Welches CI-Nutzungsverhalten zeigen diese Kinder, und welchen Einfluss nimmt es auf sprachproduktive Leistungen? Findet sich ein Zusammenhang zwischen der Frequenz der CI-Anpassungen und den sprachproduktiven Leistungen?

## Material und Methoden

Die vorliegende Datenanalyse erfolgte retrospektiv, es wurde ein positives Ethikvotum (Nr. 199_17 Bc) der Ethik-Kommission der Friedrich-Alexander-Universität Erlangen-Nürnberg eingeholt.

### Stichprobe

Eingeschlossen wurden 34 konnatal gehörlose bzw. an Taubheit grenzend schwerhörige Kinder, welche bilateral mit CI versorgt wurden. Der Versorgungszeitpunkt – definiert als Zeitpunkt der Erstaktivierung des ersten CI bei sequenzieller Implantation (*n* = 29) bzw. beider CI bei simultaner Implantation (*n* = 5) – lag zwischen dem sechsten und zwölften Lebensmonat (*M* = 8,8; *SD* = 1,7). Bei sequenzieller Versorgung erfolgte die Erstaktivierung des zweiten CI mit einem zeitlichen Abstand von 2–9 Monaten (*M* = 3,9; *SD* = 1,7) zum Zeitpunkt der Erstversorgung.

Ausschlusskriterien waren unilaterale CI-Versorgung, bimodale Versorgung und Single Sided Deafness (SSD) zugunsten einer Homogenität der Kohorte bezogen auf die Versorgungsart und den präoperativen und kontralateralen Hörstatus. Weitere Ausschlusskriterien waren das Vorliegen von cochleären Malformationen sowie entwicklungsrelevanten Syndromen und Komorbiditäten. Die Tab. 1 zeigt die Stichprobendaten im Überblick.

**Table 1:** 

Variablen		*n*	*M* (*SD*)
CI-Versorgungsalter (in Monaten)	Gesamtzahl *N*	34	8,8 (1,7)
	CI bilateral simultan	5	9,0 (1,4)
	CI bilateral sequenziell	29	8,7 (1,7)
Zeit zwischen 1. CI und 2. CI (in Monaten)		29	3,9 (1,7)
Lebensalter zum Testzeitpunkt ELFRA‑2 (in Monaten)		31	24,1 (1,0)
Lebensalter zum Testzeitpunkt SETK‑2 (in Monaten)		26	29,7 (4,0)
Geschlecht	Weiblich	11	–
	Männlich	23	

### Verfahren zur Erfassung von sprachproduktiven Leistungen

Die in der klinischen Routine erhobenen Daten der Erhebungsverfahren ELFRA‑2 und SETK‑2 wurden retrospektiv analysiert.

#### ELFRA-2.

Der ELFRA‑2 [15] wird zum Lebensalterszeitpunkt 24 Monate durchgeführt und misst mittels Elternurteil die Sprachproduktion anhand von 3 Skalen. Die erste Skala erfasst den *produktiven Wortschatz (PW)*, die Skalen *Syntax (Syn)* und *Morphologie (Mor)* erfassen die Quantität und Qualität von Mehrwortäußerungen. Die erreichten Rohwerte werden mit normierten kritischen Werten verglichen; diese liegen für *PW* bei 50, *Syn* bei 7 und *Mor* bei 2. Für 31 Kinder (91 %) lagen vollständige Datensätze des ELFRA‑2 vor, das Lebensalter zum Testzeitpunkt lag in der vorliegenden Studie bei *M* = 24,1 (*SD* = 1,0) Monaten.

#### SETK-2.

Der SETK‑2 [16] als standardisiertes Testverfahren umfasst 4 Subtests zur Sprachrezeption und -produktion, in der vorliegenden Studie wurde sich auf die Subtests *Produktion Wörter* (*P1*) und *Produktion Sätze *(*P2*) begrenzt. Die erzielten Rohwerte werden in altersrelationierte standardisierte T‑Werte mit einem Normbereich von 40–60 transformiert. Normdaten liegen für 2 Altersgruppen 24–29 und 30–35 Monate vor. Das Lebensalter zum Testzeitpunkt mit dem SETK‑2 lag in der vorliegenden Studie bei *M* = 29,7 (*SD* = 4,0) Monaten. Insgesamt konnten 26 Datensätze (76 %) in die vorliegende Analyse inkludiert werden.

### Daten zur CI-Anpassung

Es erfolgte eine systematische Auszählung der stattgefundenen CI-Anpasssitzungen. Um die Anzahl der CI-Anpassungen mit den Sprachleistungen in Bezug setzen zu können, wurde zunächst für jedes Kind der Zeitraum in Monaten zwischen CI-Versorgungszeitpunkt und den Testzeitpunkten ermittelt. Daraus erfolgte die Berechnung der CI-Anpasssitzungen pro Monat, der somit ermittelte Quotient stellt sich nun als unabhängig vom Versorgungszeitpunkt dar. Für den Testzeitpunkt ELFRA‑2 ergaben sich gemittelt über die Stichprobe 1,1 (*SD* = 0,2) CI-Anpasssitzungen pro Monat; 0,9 (*SD* = 0,2) für den Testzeitpunkt SETK‑2.

### Daten zum CI-Nutzungsverhalten

Mittels der Programmierung einer individuellen SQL-Abfrage mit MATLAB (The Mathworks Inc., Natick, MA, USA) und der Nucleaus MATLAB Toolbox (Cochlear Ltd., Sydney, NSW, Australia) wurden die Datalogeinträge der Kinder mit Cochlear-Nucleus-6- und -Nucleus-7-Systemen (*n* = 23) aus der Datenbank retrospektiv extrahiert. Ein Logeintrag ergibt sich aus 2 aufeinanderfolgenden Kopplungen des CI-Prozessors mit der Programmiersoftware. Es wurden insgesamt 1790 Logeinträge gesichtet. Sondiert wurden zunächst die Log-Daten zur durchschnittlichen täglichen CI-Nutzungsdauer in Stunden (*CoilOn-Time*) sowie die durchschnittliche tägliche Anzahl der Kontaktabbrüche der Sendespule des Prozessors zum Implantat (*CoilOff*). Die *CoilOn-Time* wird unterteilt in 5 Zeiten, die jeweils einer bestimmten akustischen Umgebung entsprechen. Diese Szenenklassifikation geschieht automatisch, sobald der Prozessor eingeschaltet ist und Kontakt zum Implantat hat, und wird im Prozessor gespeichert. Bei jeder Anpasssitzung werden diese Daten ausgelesen und in der Anpasssoftware gespeichert. Die Szenenklassifikation der akustischen Umgebung ist ein aus der Hörgerätetechnik etabliertes Verfahren, um die aktuelle Einstellung zu modifizieren [7]. Dabei wird jeweils über mehrere Sekunden der aktuelle Schallpegel, der Modulationsgrad und die Frequenzzusammensetzung von dem Hörsystem analysiert. Für das Nucleus-6/7-CI-System werden 5 Klassen von akustischer Umgebung gebildet [24]:

1. Niedriger Schallpegel: Ruhe (*Quiet*)
2. Mittlerer Schallpegel und deutliche Modulationen im Signal: Sprache (*Speech in Quiet* *=* *SiQ*)
3. Hoher Schallpegel mit mäßiger Modulation: Sprache im Störgeräusch (*Speech in Noise* *=* *SiN*)
4. Hoher Schallpegel und geringe Modulationen: Lärm (*Noise*)
5. Mittlerer oder hoher Schallpegel mit deutlichen tonalen Anteilen: Musik (*Music*)

Die für die Berechnungen verwendeten Log-Daten wurden auf nur eine CI-Seite begrenzt: Es wurde diejenige CI-Seite bestimmt, welche zum Erhebungszeitpunkt ELFRA‑2 die höhere *CoilOn-Time* aufwies. Die Log-Daten wurden zudem separat für die beiden Messzeitpunkte zur Durchführung des ELFRA‑2 und des SETK‑2 ermittelt.

### Datenauswertung und Statistik

Die statistische Auswertung erfolgte mittels IBM SPSS Statistics 24 (SPSS Inc., Chicago/IL, USA), die Erstellung der Grafiken mittels SigmaPlot 12.3 (Systat Software Inc., San Jose/CA, USA). Die Daten zu den sprachproduktiven Leistungen im ELFRA‑2 und SETK‑2 wurden unter deskriptiven Gesichtspunkten betrachtet. Vergleichsanalysen zu CI-Nutzungsverhalten und CI-Anpassungen erfolgten mittels nichtparametrischer Verfahren aufgrund der nicht normalverteilten Daten; die Prüfung auf Normalverteilung erfolgte mittels Shapiro-Wilk-Test. Korrelationsberechnungen erfolgten mittels Spearman-Rangkorrelationen, ergänzend wurden univariate lineare Regressionsanalysen durchgeführt. Das Signifikanzniveau wurde für alle statistischen Verfahren auf 5 % gesetzt, bei multivariaten Verfahren wurde die Signifikanz mittels Bonferroni-Korrektur angepasst. Abgebildete Boxplots zeigen Median und Perzentile. Der Interquartilsabstand (Box) definiert das 25. und das 75. Perzentil, die Fehlerbalken das 10. bzw. 90. Perzentil, Ausreißer liegen jeweils darunter bzw. darüber.

## Ergebnisse

Zunächst werden die Sprachleistungen und die Ergebnisse zu CI-Anpassung und CI-Nutzungsverhalten dargestellt. Es folgen die Ergebnisse der Korrelations- und Regressionsanalysen.

### Sprachproduktive Leistungen im dritten Lebensjahr

**Figure 1:**
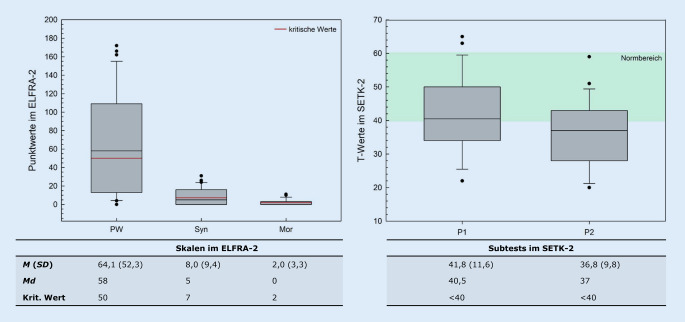

Die Kinder der untersuchten Stichprobe erreichten im Mittel die kritischen Werte der hörgesunden Normierungsstichprobe des ELFRA‑2. In der Skala *PW *wurde der kritische Wert von 50 deutlich überschritten (*M* = 64,1; *SD* = 52,3). Insgesamt zeigt sich für die Leistungen auf Wortebene eine breite Streuung; rund 55 % überschritten den kritischen Wert. In der Skala *Syn* wurde der kritische Wert von 7 knapp überschritten (*M* = 8,0; *SD* = 9,4); insgesamt erreichten bzw. überschritten rund 42 % den kritischen Wert. In der Skala *Mor *wurde der kritische Wert von 2 genau erreicht (*M* = 2,0; *SD* = 3,3); rund 32 % erreichten bzw. überschritten den kritischen Wert. Im Subtest *P1* des SETK‑2 erzielte die Stichprobe ein Ergebnis im unteren Normbereich (*M* = 41,8; *SD* = 11,6); rund 54 % lagen innerhalb des Normbereichs oder darüber. Im Subtest *P2* lagen dagegen rund 57 % unterhalb des Normbereichs (M = 36,8; *SD* = 9,8). Für beide Subtests zeigt sich eine ähnliche Streuung der erzielten T‑Werte. Die Abb. 1 zeigt die Ergebnisse in der Zusammenfassung.

### CI-Anpassung und Nutzungsverhalten

Der Zeitraum zwischen Erstanpassung des ersten CI bzw. beider CI bei simultaner bilateraler Versorgung und Testung betrug für den ELFRA‑2 im Mittel 12,4 (*SD* = 3,4) und 19,1 (*SD* = 10,7) Monate für den SETK‑2. Pro Monat ergaben sich für jedes Kind gemittelt 1,1 (*SD* = 0,2) CI-Anpassungen bezogen auf den ELFRA‑2 und 1,0 (*SD* = 0,2) CI-Anpassungen bezogen auf den SETK‑2. Die Anzahl der CI-Anpassungen pro Monat nahm zwischen den Testzeitpunkten signifikant ab (Wilcoxon-Vorzeichen-Rangtest: *Z* = −3,64; *p* < 0,0001).

Die Gesamtanzahl aller ausgelesenen Logeinträge betrug 376 für den ELFRA‑2, je Kind im Mittel 16,4 (*SD* = 4,4). *Die CoilOn-Time* lag bei 8,2 h (*SD* = 1,5 h) täglich, die Anzahl der *CoilOff* bei 62,3 (*SD* = 32,5) täglich. Bezogen auf den SETK‑2 war erwartungsgemäß die Anzahl der Logeinträge gesamt (*n* = 503) sowie gemittelt pro Kind (*M* = 21,7; *SD* = 5,5) größer aufgrund des zeitlich größeren Abstands der Testung zur Erstanpassung (Wilcoxon-Vorzeichen-Rangtest: *Z* = 3,83; *p* ≤ 0,0001). Die gemittelte tägliche *CoilOn-Time* erhöhte sich zwischen den Testzeitpunkten deskriptiv geringfügig, im Wilcoxon-Vorzeichen-Rangtest erwies sich dieser Anstieg als signifikant (*Z* = 2,93; *p* = 0,003). Die Anzahl der täglichen *CoilOff* (*M* = 59,2; *SD* = 29,5) reduzierte sich ebenfalls signifikant (Wilcoxon-Vorzeichen-Rangtest: *Z* = −2,50; *p* = 0,012). Ein Überblick der Werte ist Tab. 2 zu entnehmen.

**Table 2:** 

Testverfahren	Zeitraum Erstanpassung bis Testung (Monate)	CI-Anpassungen gesamt (*n*)	CI-Anpassungen pro Monat (*n*)	Logeinträge gesamt (*n*)	CoilOn-Time (h/Tag)	CoilOff (*n*/Tag)
**ELFRA‑2 **(*n* = 31)						
*M* (*SD*)	12,4 (3,4)	16,9 (3,5)	1,1 (0,2)	16,4 (4,4)	8,2 (1,5)	62,3 (32,5)
∑ Gesamt *n*	–	525	34,1	376	–	–
**SETK‑2 **(*n* = 26)						
*M* (*SD*)	19,1 (10,7)	19,5 (4,3)	1,0 (0,2)	21,7 (5,5)	8,4 (1,5)	59,2 (29,5)
∑ Gesamt *n*	–	526	25,8	503	–	–

**Figure 2:**
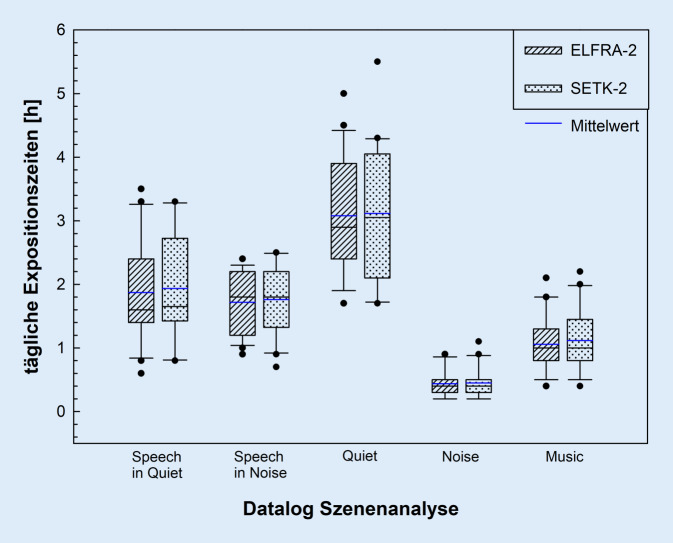

Die Auswertung der szenenbezogenen Expositionszeiten (Abb. 2) ergab, dass sich die Kinder am längsten in der Szene *Quiet* befanden, die Dauer war für den ELFRA‑2 (*M* = 3,1, *SD* = 1,0) und den SETK‑2 (*M* = 3,1; *SD* = 1,1) annähernd gleich. Die geringste Zeit verbrachten die Kinder in der Szene *Noise*; auch hier deckten sich die Werte des ELFRA‑2 (*M* = 0,4; *SD* = 0,2) und des SETK‑2 (*M* = 0,5; *SD* = 0,2). Die verbleibende Zeit verteilte sich weitestgehend gleichmäßig auf die Szenen *SiQ* (gleiche Werte für ELFRA‑2 und SETK-2: *M* = 1,9; *SD* = 0,8), *SiN* (*M* = 1,7; *SD* = 0,5 und *M* = 1,8; *SD* = 0,6) und *Music* (*M* = 1,1; *SD* = 0,4 und *M* = 1,1; *SD* = 0,5). Die mittels zweifaktorieller Varianzanalyse nach Friedmann (mit Bonferroni-Korrektur) berechneten paarweisen Vergleiche ergaben keine Veränderungen der Expositionszeiten zwischen den beiden Testzeitpunkten ELFRA‑2 und SETK‑2 bezogen auf die verschiedenen Szenen (*Z* = −0,209 bis *Z* = −0,888;* p* ≥ 0,375).

### Zusammenhang zwischen CI-Anpassung, CI-Nutzungsverhalten und Sprachleistungen

Die Sprachleistungen wurden mittels Rangkorrelationen nach Spearman auf Zusammenhänge mit Parametern des CI-Nutzungsverhaltens und der CI-Anpassung geprüft (Tab. 3). Es ergaben sich signifikante positive Korrelationen zwischen Sprachleistungen und *CoilOn-Time* (*rho* = 0,47 bis* rho* = 0,62; *p* ≤ 0,037), mit Ausnahme des Subtest *P2* im SETK‑2 (*rho* = −0,01; *p* = 0,959). Keine Zusammenhänge konnten für die Anzahl der *CoilOff* (*rho* = −0,22 bis *rho* = −0,03; *p* ≥ 0,317) und CI-Anpassungen pro Monat (*rho* = 0,09 bis *rho* = 0,25; *p* ≥ 0,221) gefunden werden. Die Analyse der Expositionszeiten ergab signifikante Korrelationen zwischen der Szene *SiQ* und Leistungen auf Wortebene sowohl in der Skala *PW* des ELFRA‑2 (*rho* = 0,65; *p* = 0,001) als auch im Subtest *P1* des SETK‑2 (*rho* = 0,60; *p* = 0,006) sowie auf Satzebene in der Skala *Syn* des ELFRA‑2 (*rho* = 0,57; *p* = 0,005). Für die Leistungen auf Satzebene ergaben sich zudem signifikante Zusammenhänge zwischen der Skala *Syn* des ELFRA‑2 und der Szene *SiN* (*rho* = 0,49; *p* = 0,017) sowie zwischen der Skala *Mor* des ELFRA‑2 und der Szene *Quiet* (*rho* = 0,53; *p* = 0,010). Die Szenen *Noise* und *Music* korrelierten mit keiner Skala bzw. keinem Subtest signifikant (*rho* = −0,23 bis *rho* = 0,12; *p* ≥ 0,346).

**Table 3:** 

	CI-AP pro Monat	CoilOn-Time	CoilOff	Speech in Quiet	Speech in Noise	Quiet	Noise	Music
**ELFRA‑2 PW**								
*rho* (*p*)	0,14 (0,467)	0,60^**^ (0,003)	−0,03 (0,884)	0,65^**^ (0,001)	0,29 (0,175)	0,41 (0,051)	0,03 (0,906)	0,12 (0,431)
*R*^2^ (*p*)	n. u.	0,43^**^ (0,001)	n. u.	0,20^*^ (0,033)	n. u.	n. u.	n. u.	n. u.
**ELFRA‑2 Syn**								
*rho* (*p*)	0,14 (0,452)	0,62^**^ (0,001)	−0,17 (0,440)	0,57^**^ (0,005)	0,49^*^ (0,017)	0,31 (0,090)	0,12 (0,584)	0,12 (0,586)
*R*^*2*^ *(p)*	n. u.	0,35^**^ (0,003)	n. u.	0,17 (0,054)	0,20^*^ (0,032)	n. u.	n. u.	n. u.
**ELFRA‑2 Mor**								
*rho* (*p*)	0,17 (0,352)	0,60^**^ (0,002)	−0,22 (0,317)	0,35 (0,104)	0,37 (0,080)	0,53^**^ (0,010)	0,10 (0,665)	0,11 (0,632)
*R*^2^ (*p*)	n. u.	0,31^**^ (0,006)	n. u.	n. u.	n. u.	0,14 (0,074)	n. u.	n. u.
**SETK‑2 P1**								
*rho* (*p*)	0,25 (0,221)	0,47^*^ (0,037)	−0,17 (0,486)	0,60^**^ (0,006)	0,19 (0,432)	0,36 (0,115)	0,04 (0,881)	−0,11 (0,649)
*R*^2^ (*p*)	n. u.	0,33^**^ (0,008)	n. u.	0,26^*^ (0,023)	n. u.	n. u.	n. u.	n. u.
**SETK‑2 P2**								
*rho* (*p*)	0,09 (0,682)	−0,01 (0,959)	−0,09 (0,728)	0,16 (0,510)	−0,29 (0,227)	0,24 (0,319)	−0,23 (0,346)	−0,19 (0,441)
*R*^2^ (*p*)	n. u.	n. u.	n. u.	n. u.	n. u.	n. u.	n. u.	n. u.

*n. u.* nicht untersucht

Signifikanzangaben: ^*^p ≤ 0,05; ^**^p ≤ 0,01

**Figure 3:**
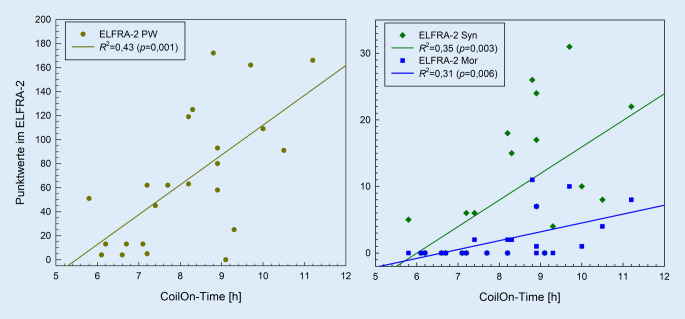

Zusätzlich wurde für alle signifikanten Spearman-Korrelationen eine univariate lineare Regression im Sinne einer Trendanalyse berechnet, um die Steigung der sprachleistungsbezogenen Variablen in Abhängigkeit von den einzelnen CI-Nutzungs‑/Expositionszeiten zu quantifizieren. Die Abb. 3 zeigt die Sprachleistungen im ELFRA‑2 in Abhängigkeit von der *CoilOn-Time*. Der Steigungsparameter (*b* = 24,85) zeigt einen Zuwachs von knapp 25 Punktwerten (entspricht 25 Wörtern) bei einer Erhöhung der *CoilOn-Time* von einer Stunde täglich. Für die Skala *Syn* zeigt sich eine Steigung von rund 4 Punktwerten (*b* = 3,99) bei einer Erhöhung der *CoilOn-Time* von einer Stunde täglich. In der Skala *Mor* beträgt dieser Zuwachs rund 1 Punktwert (*b* = 1,32).

**Figure 4:**
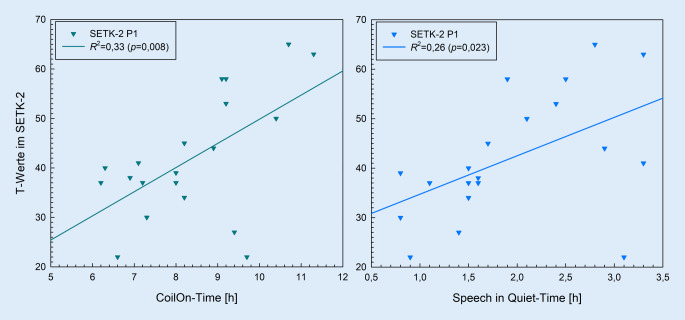

Die Abb. 4 zeigt den Einfluss der *CoilOn-Time* sowie der Einfluss der Expositionszeit in der Szene *SiQ* auf die Sprachleistungen auf Wortebene im Subtest P1 des SETK‑2. Bei einer Erhöhung der *CoilOn-Time* von einer Stunde täglich ergibt sich ein Zuwachs von rund 5 standardisierten T‑Wertpunkten (*b* = 4,88). Erhöht sich außerdem die Expositionszeit in der Szene *SiQ* um eine Stunde täglich, zeigt sich eine Steigung von rund 8 T-Wertpunkten (*b* = 7,77).

## Diskussion

### Frühe CI-Versorgung und erste Sprachproduktionsleistungen

Die Verfahren ELFRA‑2 und SETK‑2 messen erste Sprachleistungen zwischen dem zweiten und dritten Lebensjahr und differenzieren dabei zwischen Wort- und Satzebene. Insbesondere bei sehr früh CI-versorgten Kindern sind diese ersten Stadien der Lautsprachentwicklung von hohem Interesse. In der vorliegenden Studie lag das mittlere CI-Versorgungsalter bei 8,8 (*SD* = 1,7) Monaten. Die Ergebnisse zeigen, dass etwa die Hälfte der untersuchten Kinder im Alter von 2 Jahren einen lebensaltersgemäßen Wortschatz entwickelte, also den wichtigen Meilenstein der 50-Wort-Grenze [32] erreichen konnte.

Auch Boons et al. [5] fanden bei etwa der Hälfte der von ihnen untersuchten frühversorgten Kindern altersadäquate Sprachleistungen. In einer Studie von Geers et al. [14] wiesen 41 von 61 Kindern im Vorschulalter eine Sprachentwicklungsverzögerung auf. Die Datenlage zu sehr frühen Sprachleistungen ist aktuell nicht ausreichend robust, Stichproben sind häufig sehr klein zwischen den Studien heterogen, sodass weitere Studien nötig sind.

Wortschatzdefizite als solche können sich negativ auf syntaktisch-morphologische Erwerbsprozesse auswirken, da der Wortschatz als wesentlicher Prädiktor für Grammatikleistungen gilt [2, 3, 29]. In der vorliegenden Studie waren mehr als die Hälfte der Kinder in ihrer Grammatikentwicklung verzögert. Mit diesen syntaktisch-morphologischen Auffälligkeiten bei CI-Kindern befassten sich bereits zahlreiche Studien [5, 28, 30] und beschrieben Erklärungsmodelle. Boons et al. [4] zeigten darüber hinaus, dass sich eine bilaterale im Vergleich zu unilateraler CI-Versorgung günstig sowie das Vorliegen zusätzlicher Beeinträchtigungen ungünstig auf sprachproduktive Leistungen auswirken. Die vorliegende Studie inkludierte ausschließlich bilateral CI-versorgte Kinder ohne zusätzliche Beeinträchtigungen. Es kann daher angenommen werden, dass für diese Probandengruppe grundsätzlich günstige Bedingungen für den Spracherwerb bestehen. Dennoch bleibt ein großer Anteil von 45–68 % hinter den Normwerten hörgesunder Kinder zurück und weist damit eine Sprachentwicklungsverzögerung auf. Gründe hierfür könnten in psychosozial-familiären Aspekten oder auch präoperativen Versorgungsaspekten liegen, welche in der vorliegenden Studie nicht näher betrachtet wurden. Zukünftige Analysen sollten daher verstärkt diese Aspekte mit einbeziehen.

### Quantität und Qualität der CI-Anpassung

Die Grundlage für ein adäquates Hören und damit für den Spracherwerb bildet zweifelsohne eine möglichst optimale Programmierung des CI-Systems, welche individuell für jedes Kind erfolgt. Hierbei kommen subjektive und objektive Verfahren zum Einsatz, die an den kindlichen Entwicklungsstand sowie die Kooperationsfähigkeit des Kindes angepasst werden müssen [21]. Die Überprüfung eines möglichen Einflusses der CI-Anpassung auf kindliche Sprachleistungen anhand der Anzahl der CI-Anpasssitzungen ergab keinen Zusammenhang. Infolge des retrospektiven Designs der Analyse muss davon ausgegangen werden, dass die CI-Einstellungen bei Kindern mit besonders hohem Therapiebedarf auch häufiger angepasst wurden, während Kinder mit raschem Fortschritt der Hör‑/Sprachentwicklung eventuell seltener CI-Anpassungen erfahren haben. Rückschlüsse darauf, dass die Häufigkeit der CI-Anpassungen für den kindlichen Spracherwerb nicht von Bedeutung ist, können daher nicht gezogen werden. Vielmehr sollten zukünftige Studien qualitative Aspekte der Programmierung (z. B. Hörschwellen, Behaglichkeitsschwellen, Dynamikbereich) berücksichtigen.

### Einfluss der täglichen CI-Nutzungsdauer auf frühe sprachproduktive Leistungen

Die Auswertung der Logeinträge ergab eine mittlere *CoilOn-Time* von 8,2–8,4 h täglich, diese ist mit den Werten der Kleinkindergruppen der Studien von Oberhoffner et al. [27] (*Md* = 8,8 h) und Busch et al. [8] (*Md* = 8,5 h) vergleichbar. Es konnte zudem gezeigt werden, dass bereits innerhalb eines knappen halben Jahres die tägliche *CoilOn-Time* signifikant anstieg, gepaart mit einer signifikanten Reduktion der Anzahl an täglichen *CoilOff*. Beides lässt sich auf das zunehmende Lebensalter der untersuchten Kinder zurückführen. Auch Easwar et al. stellten eine Reduzierung der *CoilOff* mit zunehmendem Lebensalter der Kinder fest [11].

Die Analysen zeigen, dass sich eine hohe CI-Nutzungsdauer positiv auf die Sprachproduktion auswirkt. Die Bedeutung für die Wortproduktion scheint dabei besonders hoch zu sein: Für die Stichprobe zeigte sich im verwendeten einfachen Regressionsmodell ein Anstieg von rund 25 Wörtern zum 24. Lebensmonat pro Erhöhung der täglichen *CoilOn-Time* um eine Stunde. Das Ausmaß dieses Zuwachses wird besonders deutlich, betrachtet man die kritische Grenze von 50 gesprochenen Wörtern, welche bei hörgesunden 2‑jährigen Kindern als Erwartungswert gilt. Aber auch die Grammatikleistung wird bei den untersuchten Kindern begünstigt durch eine höhere tägliche *CoilOn-Time*. Belegt werden kann dies jedoch nur für die mit dem ELFRA‑2 ermittelten Leistungen, die Ergebnisse des Subtest *P2* des SETK‑2 bestätigen dies nicht. Als mögliche Erklärung hierfür kann vermutet werden, dass der Subtest *P2* die Leistungen weniger differenziert erfasst als die Skalen *Syn* und *Mor* des ELFRA‑2.

### Szenenanalyse und Sprachleistungen

Von besonderem Interesse ist der zeitliche Umfang der Sprachexposition (*SiQ* und *SiN*). Erwartungsgemäß erwies sich die tägliche Expositionszeit in der Szene *SiQ* als ein signifikanter Einflussfaktor für sprachproduktive Leistungen, insbesondere auf Wortebene. Damit entsprechen die vorliegenden Befunde denen von Guerzoni und Cuda [17], welche einen signifikanten Zusammenhang zwischen dem lexikalischen Quotienten und der kumulierten Expositionszeit in der Szene *SiQ* bei CI-versorgten Kindern feststellten. Folglich begünstigt die Quantität des Sprachinputs die frühkindliche Sprachproduktion; mögliche Auswirkungen der Qualität des Sprachinputs müssen an dieser Stelle unberücksichtigt bleiben. Weitere Studien mit qualitativen Analysen könnten jedoch zu einem Erklärungsmodell für die besseren syntaktischen Sprachleistungen bei höherer Expositionszeit in der Szene *SiN* führen.

Kritisch betrachtet werden muss das Verhältnis der Expositionszeiten in den Szenen *Quiet* und *SiQ*. Einerseits lässt sich die hohe Ruheexposition anhand des jungen Alters der Kinder erklären. Säuglinge und Kleinkinder halten sich in der Regel die meiste Zeit in häuslicher Umgebung auf, welche eher von geringem Lärmpegel geprägt ist. Andererseits ist zu beachten, dass die Szene *Quiet* nicht zwingend absoluter Ruhe entsprechen muss. Vielmehr können hier auch Geräusch- und Sprachanteile vorhanden sein, welche jedoch zeitlich zu kurz ausfallen, als dass sie im Rahmen der automatischen Szenenklassifikation als Veränderung der Hörumgebung erkannt werden. Nach Mauger et al. [24] benötigt das CI-System mehrere Sekunden, um anhand einer Merkmalsextraktion die akustische Situation zu klassifizieren. Demnach ist es möglich, dass Interaktionssituationen zwischen Kind und Elternteil nicht als Sprachexposition gewertet werden, da diese Situationen häufig durch kurze prägnante Sprachäußerungen (statt kontinuierlichem Sprachinput), längere Sprechpausen sowie reine Handlungsphasen charakterisiert sind. Dieser Erklärungsansatz kann auch für die Betrachtung des ermittelten Zusammenhangs zwischen den morphologischen Sprachleistungen und der Szene *Quiet* herangezogen werden. Demnach ist anzunehmen, dass ein niedriger Hintergrundpegel in Verbindung mit prägnanten Sprachangeboten die Sensitivität für leise Sprachanteile erhöht und somit die Perzeption von Morphemen verbessert. Dies wird gestützt durch die *Morpheme-in-Noise-Perception-Deficit(MIND)-Hypothese* nach Hammer [18], nach welcher die Perzeption grammatischer Morpheme bei CI-versorgten Kindern insbesondere in Umgebungen mit Störschall eingeschränkt ist.

## Ausblick

Kinder, die innerhalb des ersten Lebensjahrs bilateral mit CI versorgt werden, können bereits in Stadien des frühen Lautspracherwerbs einen altersgemäßen Stand erreichen. Eine sehr frühe CI-Versorgung ist daher anzustreben. Durch die vorliegende Analyse konnte gezeigt werden, dass der Lautspracherwerb beeinflusst wird durch die tägliche CI-Nutzungsdauer und Sprachexposition. Frühere Studien stützen sich auf subjektive Elternangaben zum kindlichen CI-Nutzungsverhalten, heute ermöglicht das systemintegrierte Datalogging eine objektive Erfassung und damit eine verbesserte Beurteilbarkeit des CI-Nutzungsverhaltens. Zudem bieten sich dadurch erweiterte Auswertungsmöglichkeiten, auch im Kontext kindlicher Hör- und Sprachleistungen. Die dadurch gewonnenen Erkenntnisse sollten unmittelbar in die praktische Elternberatung im Rahmen der postoperativen CI-Rehabilitation einfließen. Die Hörbedingungen im Alltag des Kindes können dadurch optimiert und der Entwicklungsrahmen begünstigt werden. Zukünftige Studien sollten unter Einbezug weiterer potenzieller hör- und sprachrelevanter Einflussfaktoren geplant werden.

## Fazit für die Praxis

- Gehörlose Kinder, die innerhalb des ersten Lebensjahres bilateral mit CI versorgt werden, können bereits im dritten Lebensjahr einen lebensaltersgemäßen produktiven Sprachentwicklungsstand zeigen.
- Je höher die tägliche CI-Nutzungsdauer und Sprachexposition bei Kleinkindern mit CI ist, desto mehr lautsprachliche Äußerungen werden produziert.
- Datalogging bietet die Möglichkeit, Nutzungsdaten unabhängig vom Elternurteil auszuwerten und die Ergebnisse in die postoperative CI-Rehabilitation und Elternberatung einfließen zu lassen.
